# Probiotics in 14-day triple therapy for Asian pediatric patients with *Helicobacter pylori* infection: a network meta-analysis

**DOI:** 10.18632/oncotarget.21633

**Published:** 2017-10-07

**Authors:** Juanjuan Wen, Pailan Peng, Pengfei Chen, Lirong Zeng, Qinghua Pan, Wenbin Wei, Jianhua He

**Affiliations:** ^1^ Department of Gastroenterology, The Central Hospital of Enshi Autonomous Prefecture, Enshi, China; ^2^ Department of Gastroenterology, Renmin Hospital of Wuhan University, Wuhan, China; ^3^ Department of Gastroenterology, Zhongnan Hospital of Wuhan University, Wuhan, China

**Keywords:** Helicobacter pylori, probiotics, pediatrics, 14-day triple therapy, network meta-analysis

## Abstract

Although 14-day triple therapy has been widely administrated for eradicating *Helicobacter pylori (H. pylori)* in Asia, its antibiotic-associated side effects restrict the effectivity of eradication therapy in pediatric patients. Therefore, a network meta-analysis (NMA) was conducted to compare the efficacy and safety of probiotics supplemented in 14-day triple therapy in Asian pediatric patients.

**Materials and Methods:**

Randomized controlled trials (RCTs) were retrieved comprehensively in electronic databases (such as PubMed, Cochrane library, Embase, CNKI, Wan fang database, VIP database and CBM) until April 2017. Additional references were obtained from reviewed articles. NMA was performed using a random-effects model under a frequentist framework.

**Results:**

Seventeen RCTs were included. NMA indicated that *Bifidobacterium infantis+Clostridium butyricum* was most beneficial for *H. pylori* eradication rates (P-score = 0.82) and *Bacillus mesentericus*+*Clostridium butyricum*+*Streptococcus faecalis* for total side effects (P-score = 0.77). Taken together, Bacillus mesentericus+Clostridium butyricum+Streptococcus faecalis was the best one to supplement in 14-day triple therapy due to its efficacy (P-score = 0.72) and safety (P-score = 0.77). Additionally, pairwise meta-analysis indicated that probiotics supplemented 14-day triple therapy significantly increased *H. pylori* eradication rates (RR: 1.16, 95%CI: 1.07–1.26) and reduced the incidence of total side effects (RR: 0.40, 95%CI: 0.34–0.48) compared with placebo.

**Conclusions:**

*Bacillus mesentericus+Clostridium butyricum+Streptococcus faecalis* is the optimal probiotic regime of reducing total side effects and improving eradication rates when supplemented 14-day triple therapy. Further direct evidence is needed to warrant it.

## INTRODUCTION

In contrast with developed countries, *Helicobacter pylori* (*H. pylori*) infections occur earlier in life and with a higher prevalence in the developing world [1, 2]. In parts of the developing world, especially in Asian countries, the prevalence of *H. pylori* infection increased with age and exceeded 80% by 19 years of age [2]. For instance, in Bangladesh, the prevalence of *H. pylori* was already 58% by 4 years of age and quickly rose to 82% by 9 years of age [2]. Similar findings were reported from pediatric studies in other Asian countries including India, Sri Lanka and China [1, 3, 4]. Children infected with *H. pylori* could develop many complications and symptoms like recurrent abdominal pain, dyspepsia, iron deficiency anemia, chronic gastritis, peptic ulcers and gastric cancer [5]. *H. pylori* infection has also been reported to cause a wide variety of extra-digestive manifestations, such as sudden infant death syndrome and short stature [6]. *H. pylori* eradication not only can cure peptic ulcers temporarily, but also reduce the lifetime risk of gastric cancer in long term due to increasing longevity in developing countries [7].

Because *H. pylori* is a bacterium, antibiotics certainly as a therapeutic agent will be administrated to eradicate it, like recent triple therapy. Nevertheless, recently accepted triple therapies still demonstrate failure rates as high as 20% to 30% due to increasing clarithromycin and metronidazole resistances as well as poor tolerability and reduced compliance [7–9]. A longer treatment duration may increase eradication rates that suggests an increase to 14 days instead of 7 days [7]. There is a strong recommendation by Joint ESPGHAN/NASPGHAN Guidelines for 14-day triple therapy as first-line therapy for pediatric patients with *H. pylori* infection where clarithromycin and metronidazole resistances are presenting commonly in developing countries [10]. In addition, supplementary probiotics can reduce side effects and improve efficacy of *H. pylori* eradication therapies [11–16].

Although these studies suggest that there may be a benefit for reduction of side effects and improved efficacy when added to *H. pylori* eradication therapies, the evidence from Asia is limited and the same meaningful conclusions amongst these different probiotic strains seemed not be obtained as well as for 14-day triple therapy. Therefore, network meta-analysis (NMA) was performed to compare the efficacy and safety of specific probiotic supplementations in 14-day triple therapy for Asian pediatric population.

## MATERIALS AND METHODS

We performed and reported the results of this pairwise and NMA in accordance with Preferred Reporting Items for Systematic Reviews and Meta-Analyses (PRISMA) statements [17, 18].

### Literature research

We conducted a comprehensive search on databases including PubMed, Cochrane library, Embase and 4 Chinese Database [China National Knowledge Infrastructure (CNKI), database of Wan fang, VIP database and the Chinese Biomedical Database(CBM)] until April 2017. Literature were identified with keywords including (*Helicobacter pylori* OR *H. pylori* OR *HP*), probiotics, (pediatrics OR children), (randomized controlled trial OR RCT OR clinical trial) and (triple therapy OR eradication therapy) (search strategy seen in Supplementary Files). Additionally, manual search for the references cited in reviews and meta-analyses was performed as well. There were no language restrictions.

### Inclusion and exclusion criteria

We included the RCTs meeting following eligibility criteria: (i) clinical trials from Asia; (ii) pediatric patients with *H. pylori* infection; (iii) clinical trials comprising of 14-day triple therapy (proton pump inhibitor and two antibiotics) plus placebo or no extra intervention and the same triple therapy plus probiotics; (iv) *H. pylori* eradication was confirmed at least 4 weeks after therapy termination. Articles were excluded if (i) reviews or comments; (ii) articles without data of *H. pylori* eradication rates and/or side effects.

### Study selection

The articles were screened and selected by two researchers (Pengfei Chen and Lirong Zeng) independently. Initially, records of duplicates were removed using Endnote 7.4 software (Thomson Reuters). Remaining abstracts and full texts were reviewing based on inclusion and exclusion criteria. Disagreements were resolved by discussions.

### Data extraction and quality assessment

Two reviewers (Juanjuan Wen and Pailan Peng) independently extracted the relevant information from each eligible study depending on a pre-prepared data abstraction sheet. Data were including location and study design of trials, clinical characteristics and number of patients, initial and rechecking methods for conformation of *H. pylori* infection, strains and period of probiotic regimens, eradication regimens and outcome data. The quality of included studies was assessed using Jadad scale, including three items such as randomized (2 points), double-blinding (2 points) and withdrawals and drop outs (1 point) [19]. A Jadad score of 3 or higher was considered as high quality. Disagreements were resolved by discussions.

The primary outcomes were eradication rates of *H. pylori* and incidence of total side effects, and secondary outcomes included incidence of specific side effects. The specific side effects of interest were the common symptoms that appeared during treatment, including diarrhea, nausea or vomiting, taste disturbance, loss of appetite, bloating, headache, constipation, and abdominal pain.

### Data synthesis and analysis

Conventional pairwise meta-analysis was initially carried out for the comparison between probiotic group and placebo group. The estimated effects using relative ratios (RRs) with 95% confidence intervals (CIs) based on the random effects model were analyzed by STATA 11.0 software (Stata Corporation, College Station, TX, USA). Secondary, NMA based on the random effects model was performed by netmeta R package with a frequentist framework [20, 21]. The P-scores that were based solely on the point estimates and standard errors of the network estimates were calculated to rank all probiotic regimens and the higher P-score value was correspond to better result for respective probiotic regimen [22].

Publication bias was evaluated using the Egger's and Begg's tests [23, 24]. If a *P value* < 0.05, a trim and fill method was further conducted and recalculated the pooled estimate iteratively, until the funnel plot was symmetrical around the (new) pooled estimate [25]. Additionally, heterogeneity was measured with Q statistic and I^2^ statistic [26, 27]. If an I^2^ value of > 50% or a *P value* < 0.05, sensitivity analysis was conducted by excluding one individual study each time to assess the influence of each individual study on the pooled RRs.

## RESULTS

### Study characteristics

**Figure 1:**
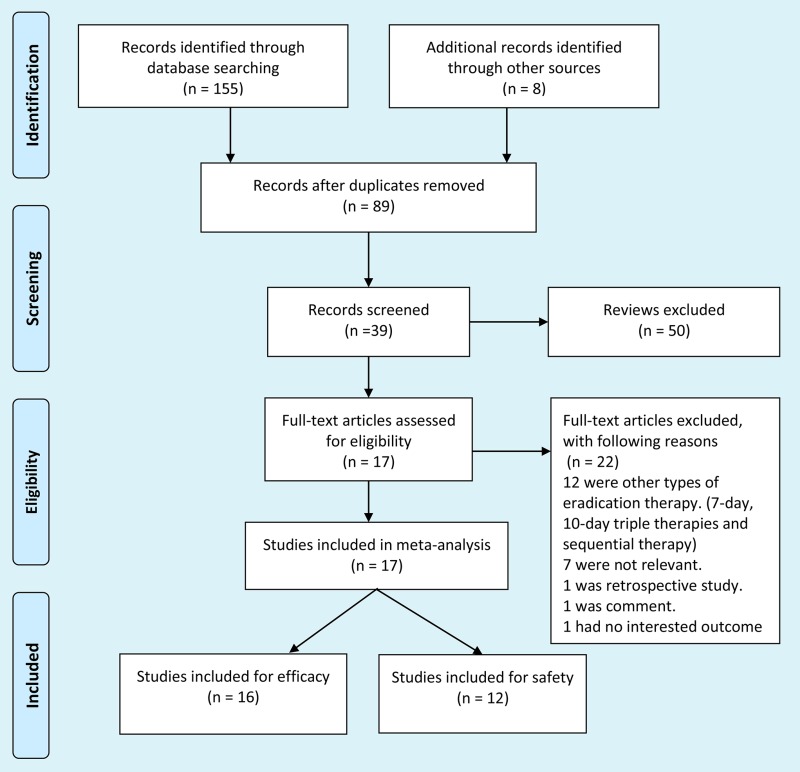
Identification process for eligible trials

A systematic search yielded a total of 213 articles. After literature reviewing, 89 duplicates were removed and 50 reviews were excluded, respectively. Additionally, 22 articles were excluded due to not met included criteria. Finally, a total of 17 trials were identified (Figure 1) and listed in Supplementary Table 5 [28–44].

The included trials involving 1932 pediatric patients has investigated 10 probiotic regimens, such as (1) *Bifidobacterium animalis* (*B. animalis*)*+Lactobacillus acidophilus*(*L. acidophilus*)*+L. casei;* (2) *Bacillus mesentericus* (*B. mesentericus*)*+Clostridium butyricum* (*C. butyricum*)*+Streptococcus faecalis* (*S. faecalis*)*;* (3) *B. bifidum+B. infantis+L. acidophilus+L. bulgaricus+L. casei+L. reuteri+ Streptococcus;* (4) *B. infantis+ C. butyricum;* (5) *B. longum+Enterococcus faecalis* (*E. faecalis*)*+L. acidophilus;* (6) *B. longum+L. bulgaricus+S. thermophilus;* (7) *L. acidophilus;* (8) *L. acidophilus+B. bifidum;* (9) *L. delbrueckii+L. acidophilus+Lactococcuslactis* (*L. lactis*)*;* (10) *Saccharomyces boulardii* (*S. boulardii*). There were 15 trials from China, 1 from Turkey and 1 from Iran. Assessing by Jadad scale, the overall Jadad scores of study quality ranged from 2 to 4 and the median of Jadad scores was 3.

### Conventional pairwise meta-analysis

**Figure 2:**
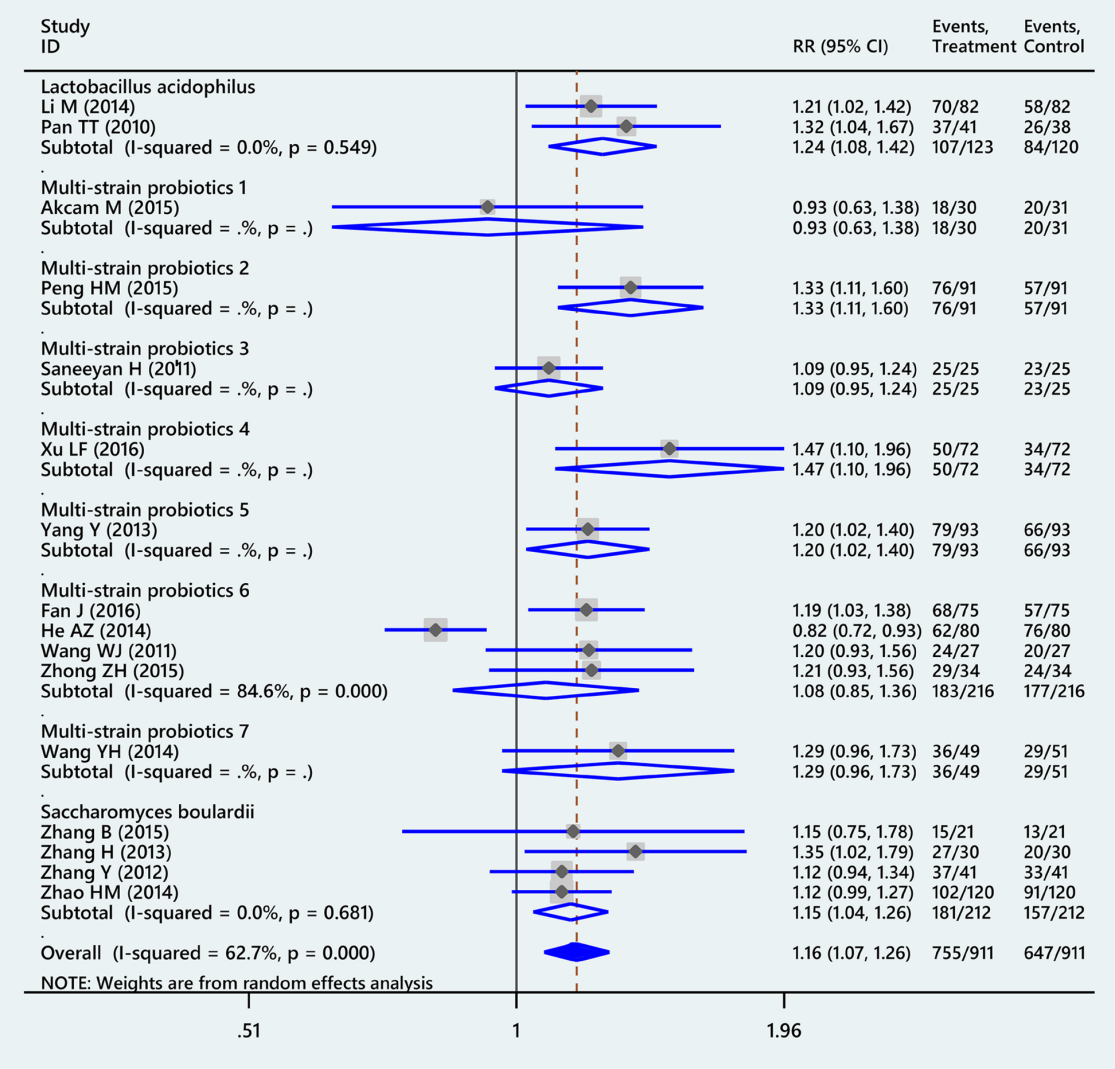
Forest plot of conventional meta-analysis for *Helicobacter pylori* eradication of probiotic regimens supplemented 14-day triple therapy compared with placebo Multi-strain probiotics 1: *Bifidobacterium animalis+ Lactobacillus acidophilus+Lactobacillus casei;* Multi-strain probiotics 2: *Bacillus mesentericus+Clostridium butyricum+Streptococcus faecalis;* Multi-strain probiotics 3: *Bifidobacterium bifidum+Bifidobacterium infantis+Lactobacillus acidophilus+Lactobasillus bulgaricus+Lactobasillus casei, Lactobasillus reuteri+Streptococcus;* Multi-strain probiotics 4: *Bifidobacterium infantis+Clostridium butyricum;* Multi-strain probiotics 5: *Bifidobacterium longum+Enterococcus faecalis+Lactobacillus acidophilus;* Multi-strain probiotics 6: *Bifidobacterium longum+Lactobacillus bulgaricus+Streptococcus thermophilus;* Multi-strain probiotics 7: *Lactobacillus acidophilus+Bifidobacterium bifidum*; Multi-strain probiotics 8: *Lactobacillus delbrueckii+Lactobacillus acidophilus+Lactococcus lactis*).

**Figure 3:**
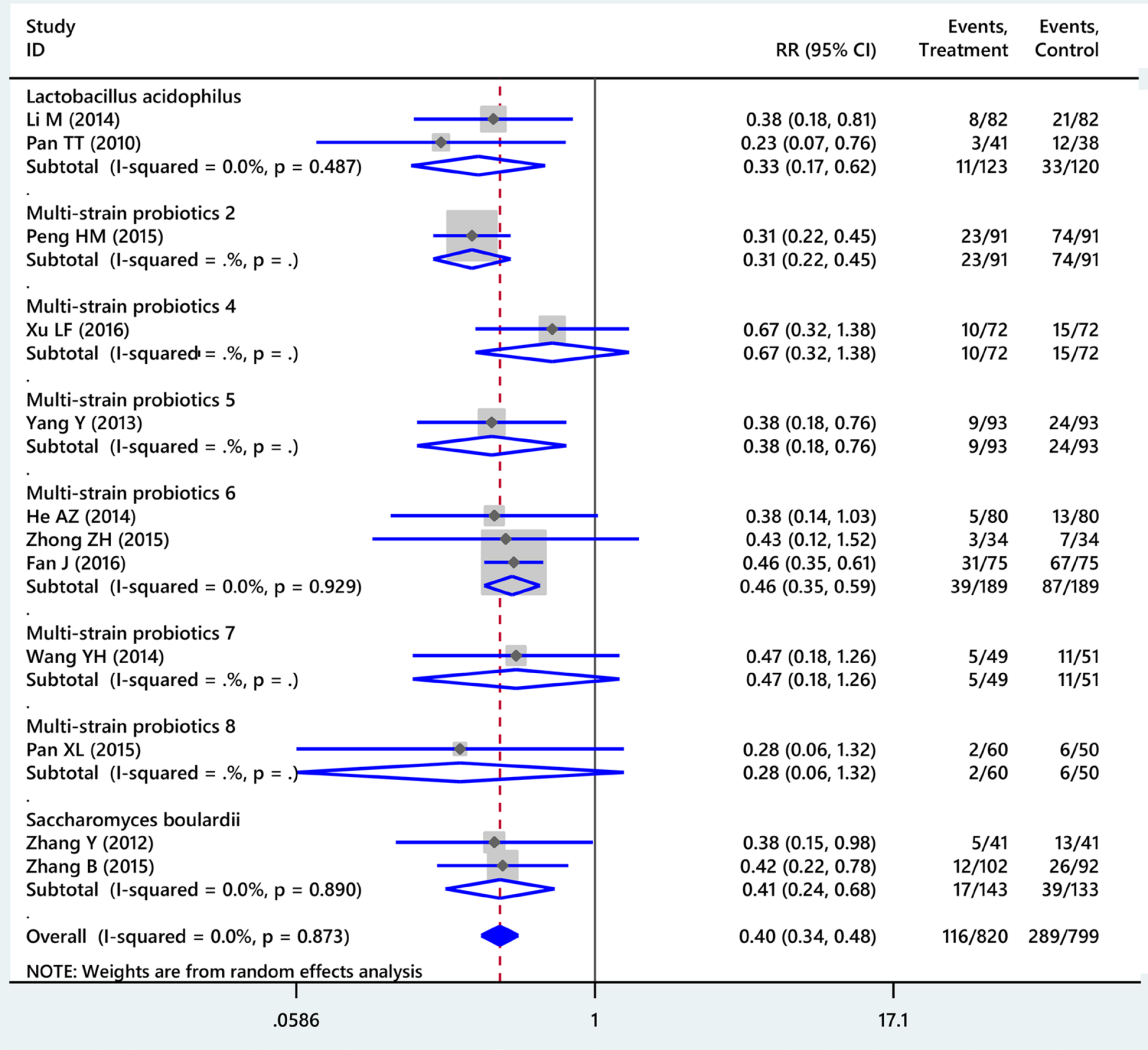
Forest plot of conventional meta-analysis for total side effects of probiotic regimens supplemented 14-day triple therapy compared with placebo Multi-strain probiotics 1: *Bifidobacterium animalis+Lactobacillus acidophilus+Lactobacillus casei;* Multi-strain probiotics 2: *Bacillus mesentericus+Clostridium butyricum+Streptococcus faecalis;* Multi-strain probiotics 3: *Bifidobacterium bifidum+Bifidobacterium infantis+Lactobacillus acidophilus+Lactobasillus bulgaricus+Lactobasillus casei, Lactobasillus reuteri+Streptococcus;* Multi-strain probiotics 4: *Bifidobacterium infantis+Clostridium butyricum;* Multi-strain probiotics 5: *Bifidobacterium longum+Enterococcus faecalis+Lactobacillus acidophilus;* Multi-strain probiotics 6: *Bifidobacterium longum+Lactobacillus bulgaricus+Streptococcus thermophilus;* Multi-strain probiotics 7: *Lactobacillus acidophilus+Bifidobacterium bifidum;* Multi-strain probiotics 8: *Lactobacillus delbrueckii+Lactobacillus acidophilus+Lactococcus lactis*).

Compared with placebo, probiotics supplemented 14-day triple therapy significantly improved eradication rates of *H. pylori* (RR: 1.16, 95% CI: 1.07–1.26, I^2^ = 62.7%) (Figure 2). After sensitivity analysis was performed by omitting the study by He AZ [32], adjusted RR was 1.19 (95% CI: 1.13–1.25, I^2^ = 0%). In term of safety, probiotics supplemented 14-day triple therapy could reduce incidence of total side effects compared with placebo (RR: 0.40, 95% CI: 0.34–0.48, I^2^ = 0%) (Figure 3). Additionally, subgroup analyses for specific probiotics found that *L. acidophilus, S. boulardii* and several multi- strain probiotics could significantly enhance the effectiveness of 14-day triple therapy (more detail seen in Supplementary Tables 1–2).

### Network meta-analysis

**Figure 4:**
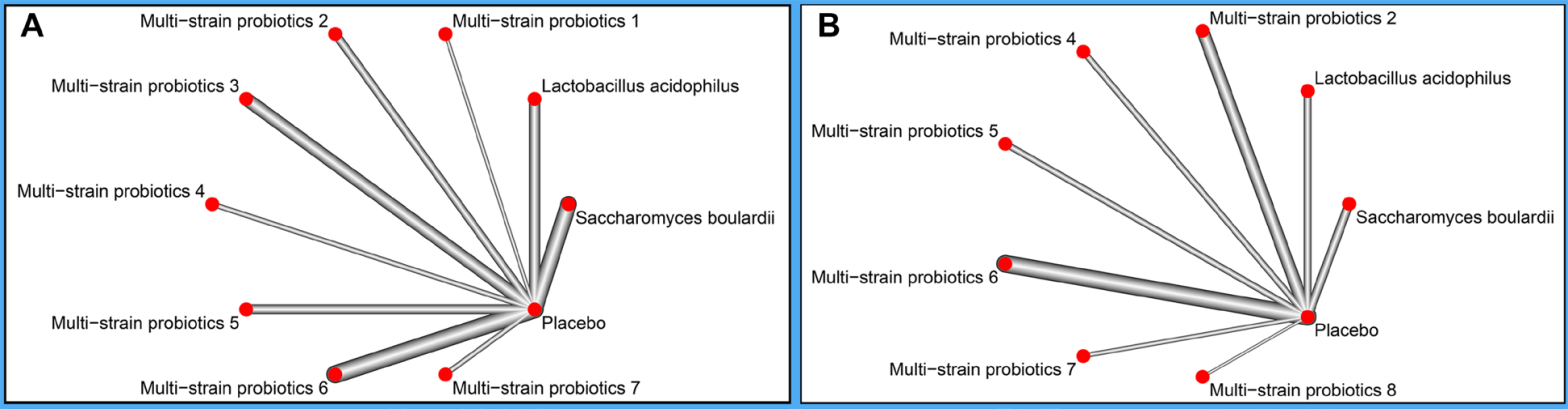
Network plots of probiotic regimens included in network meta-analysis (**A**) Network relations of probiotic regimens that supplemented 14-day triple therapy on *Helicobacter pylori* eradication. (**B**) Network relations of probiotic regimens that supplemented 14-day triple therapy on total side effects. The nodes correspond to and are labeled according to the probiotic regimens, and the edges show which probiotic regimens are directly compared. The thickness of the lines is proportional to the inverse standard error of the direct comparisons. Multi-strain probiotics 1: *Bifidobacterium animalis+Lactobacillus acidophilus+Lactobacillus casei;* Multi-strain probiotics 2: *Bacillus mesentericus+Clostridium butyricum+Streptococcus faecalis;* Multi-strain probiotics 3: *Bifidobacterium bifidum+Bifidobacterium infantis+Lactobacillus acidophilus+Lactobasillus bulgaricus+Lactobasillus casei, Lactobasillus reuteri+Streptococcus;* Multi-strain probiotics 4: *Bifidobacterium infantis+Clostridium butyricum;* Multi-strain probiotics 5: *Bifidobacterium longum+Enterococcus faecalis+Lactobacillus acidophilus;* Multi-strain probiotics 6: *Bifidobacterium longum+Lactobacillus bulgaricus+Streptococcus thermophilus;* Multi-strain probiotics 7: *Lactobacillus acidophilus+Bifidobacterium bifidum; Multi-strain probiotics 8: Lactobacillus delbrueckii+Lactobacillus acidophilus+Lactococcus lactis*).

**Figure 5:**
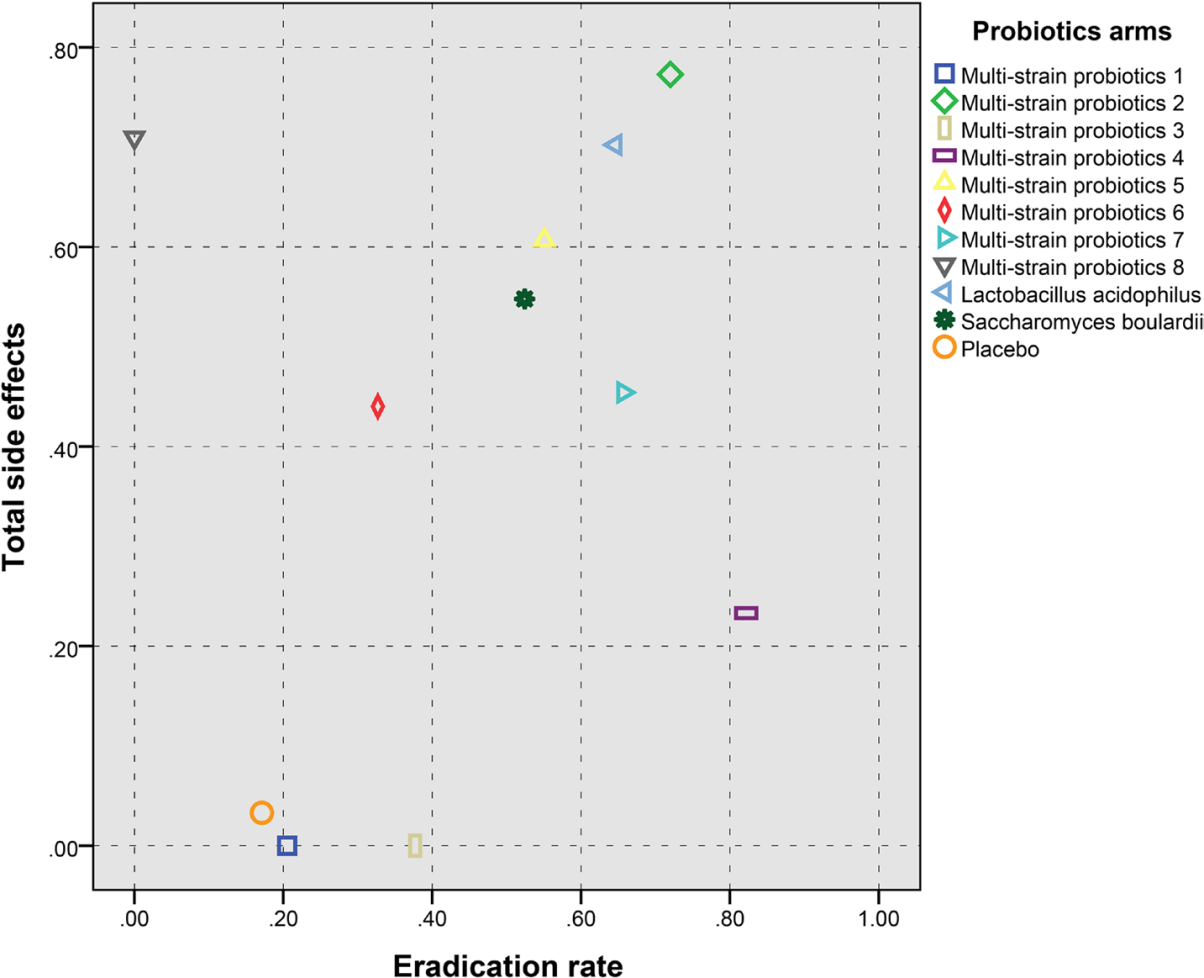
The scatter plot based on P-scores for total side effects (Y-axis) and Helicobacter pylori eradication (X-axis) of probiotic regimens supplemented 14-day triple therapy The P-scores are larger and better, and if P-score = 0, this parameter is missing. Therefore, Multi-strain probiotics 2 is the better result for the probiotic regimen supplemented 14-day triple therapy with both total side effects and *Helicobacter pylori* eradication rate. Multi-strain probiotics 1: *Bifidobacterium animalis+Lactobacillus acidophilus+Lactobacillus casei;* Multi-strain probiotics 2: *Bacillus mesentericus+Clostridium butyricum+Streptococcus faecalis;* Multi-strain probiotics 3: *Bifidobacterium bifidum+Bifidobacterium infantis+Lactobacillus acidophilus+Lactobasillus bulgaricus+Lactobasillus casei, Lactobasillus reuteri+Streptococcus;* Multi-strain probiotics 4: *Bifidobacterium infantis+Clostridium butyricum;* Multi-strain probiotics 5: *Bifidobacterium longum+Enterococcus faecalis+Lactobacillus acidophilus;* Multi-strain probiotics 6: *Bifidobacterium longum+Lactobacillus bulgaricus+Streptococcus thermophilus;* Multi-strain probiotics 7: *Lactobacillus acidophilus+Bifidobacterium bifidum;* Multi-strain probiotics 8: *Lactobacillus delbrueckii+Lactobacillus acidophilus+Lactococcus lactis*).

There were 9 and 8 probiotic regimens supplemented 14-day triple therapy reporting data of eradication rates and total side effects, respectively (Figure 4). In term of improving eradication rates of *H. pylori*, *B. infantis+C. butyricum* was the best to add in 14-day triple therapy (P-score = 0.82). *B. mesentericus+C. butyricum+S. faecalis* was most beneficial for total side effects (P-score = 0.77). Taken together, *B. mesentericus+C. butyricum+S. faecalis* was the best one to supplement in 14-day triple therapy due to its efficacy (P-score = 0.72) and safety (P-score = 0.77) (Figure 5). The relative effectiveness of all possible pairs of probiotics was presented in league tables (Supplementary Tables 3–4).

**Figure 6:**
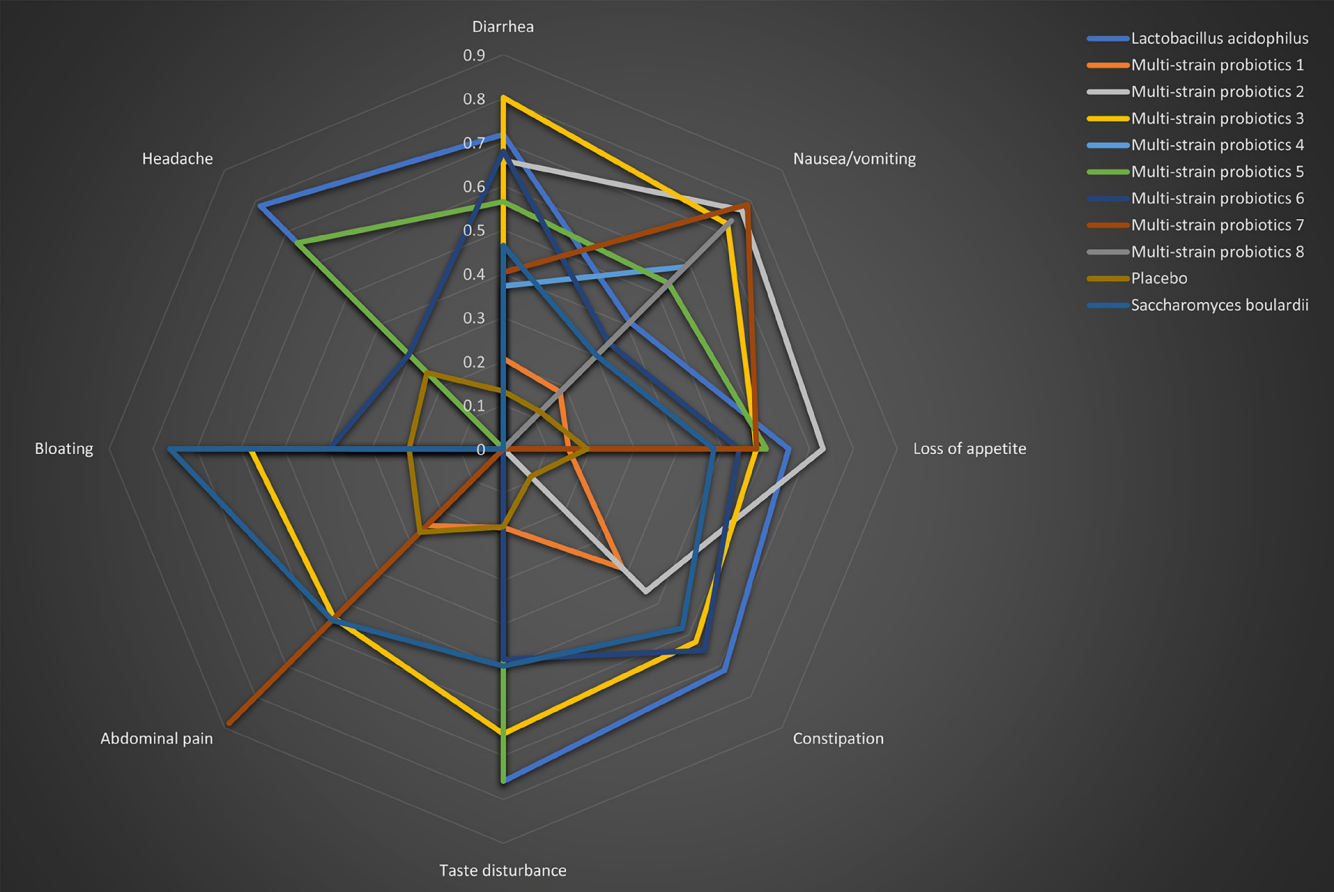
Radar chart based on P-scores for subtypes of side effects of probiotic regimens supplemented 14-day triple therapy Different probiotic regimens are shown in different colors. The axes of this radar chart are oriented in the way that the best values for a particular parameter are positioned outside. Therefore, the better results for a probiotic regimen the bigger area covered by the polygon for this probiotic regimen on this radar chart. In addition, the P-scores are larger and better, and if P-score = 0, this parameter is missing. Multi-strain probiotics 1: *Bifidobacterium animalis+Lactobacillus acidophilus+Lactobacillus casei;* Multi-strain probiotics 2: *Bacillus mesentericus+Clostridium butyricum+Streptococcus faecalis;* Multi-strain probiotics 3: *Bifidobacterium bifidum+Bifidobacterium infantis+Lactobacillus acidophilus+Lactobasillus bulgaricus+Lactobasillus casei, Lactobasillus reuteri+Streptococcus;* Multi-strain probiotics 4: *Bifidobacterium infantis+Clostridium butyricum;* Multi-strain probiotics 5: *Bifidobacterium longum+Enterococcus faecalis+Lactobacillus acidophilus;* Multi-strain probiotics 6: *Bifidobacterium longum+Lactobacillus bulgaricus+Streptococcus thermophilus;* Multi-strain probiotics 7: *Lactobacillus acidophilus+Bifidobacterium bifidum; Multi-strain probiotics 8: Lactobacillus delbrueckii+Lactobacillus acidophilus+Lactococcus lactis*).

As for subtypes of side effects, *B. bifidum+B. infantis+L. acidophilus+L. bulgaricus+L. casei+L. reuteri+ Streptococcus* was most beneficial for reducing the incidence of diarrhea (P-score = 0.80), *L. acidophilus+B. bifidum* for nausea/vomiting (P-score = 0.79), *B. mesentericus+C. butyricum+ S. faecalis* for loss of appetite (P-score = 0.73), *L. acidophilus* for constipation (P-score = 0.71), *L. acidophilus* for taste disturbance (P-score = 0.76), *L. acidophilus+B. bifidum*for Abdominal pain(P-score = 0.89), *S. boulardii* for bloating (P-score = 0.76)*, L. acidophilus* for headache (P-score = 0.78) (Figure 6).

### Publication bias

**Figure 7:**
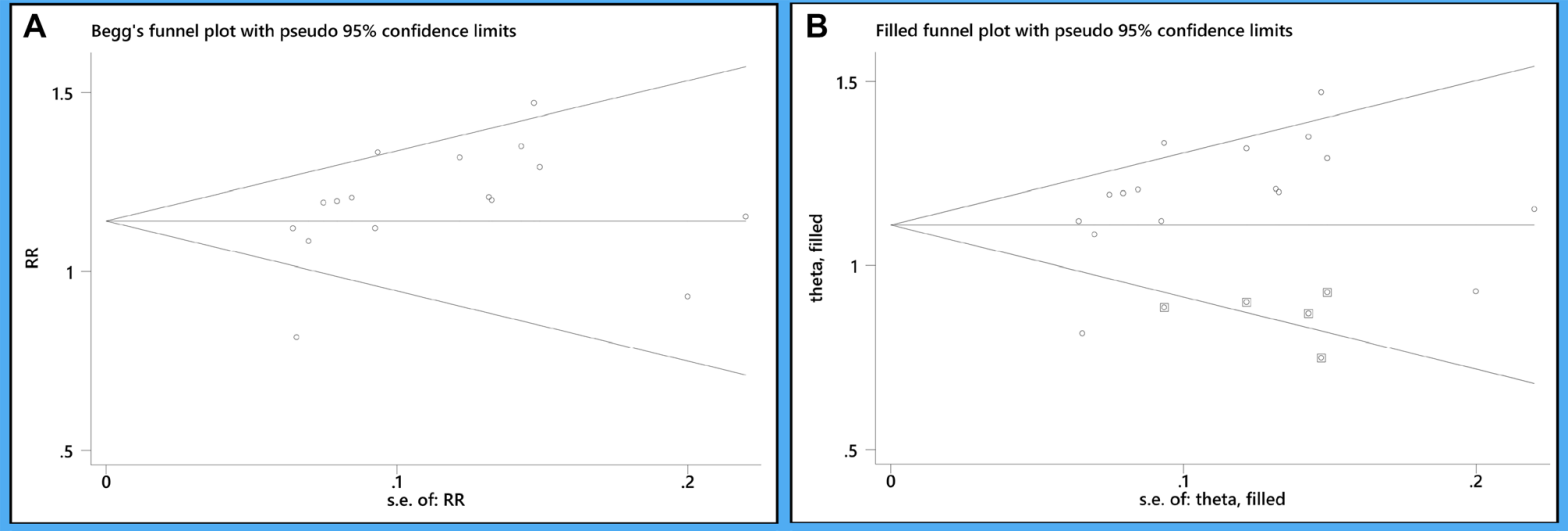
(**A**) Asymmetrical funnel plot of *H. pylori* eradication rates; (**B**) trim and fill method for previous asymmetrical funnel plot of *H. pylori* eradication rates. The squares represent the adjusted studies.

Publication bias was assessed using data of *H. pylori* eradication rates. Visual inspection of the Begg's funnel plot found a slightly asymmetrical distribution (Figure 7A), which indicated having potential publication bias (*P* = 0.08). By trimming and inputting 5 studies using the trim and fill method (Figure 7B), the recalculated pooled RR for *H. pylori* eradication rates was 1.20 (95% CI: 1.12–1.29) (Supplementary Figures 1–2), which was not significantly changed from the initial estimate (RR: 1.16, 95% CI: 1.07–1.26).

## DISCUSSION

This complexed meta-analysis included RCTs all from Asian countries, where appears high prevalence rate of *H. pylori* infection [7]. Our meta-analysis showed that probiotic supplementation can statistically benefit 14-day triple therapy with both eradication rates and safety. Amongst included probiotics, *B. infantis+C. butyricum* was best for eradication rates of *H. pylori* and *B. mesentericus+C. butyricum+S. faecalis* best for reducing the incidence of total side effects. Additionally, it further found that beneficial effects of probiotics are strain specific. For example, as an addition in 14-day triple therapy, *B. bifidum+B. infantis+L. acidophilus+L. bulgaricus+L. casei+L. reuteri+ Streptococcus* could reduce the incidence of diarrhea, which is the most common side effect associated with antibiotics. Given the results from this study, probiotics as a supplementation to 14-day triple therapy are considered as an alternative treatment regimen. Moreover, specific probiotics are administrated for corresponding specific side effect.

Probiotics, namely probiotic bacteria, have defined as live microorganisms, which most distribute in anterointernal mucosa and skin, confer a health benefit for the host. In infectious disease, probiotics act in the gastrointestinal luminal environment by reducing luminal pH, inhibiting adhesion of other harmful microorganisms and antagonizing pathogenic bacteria by secreting bacteriocins [45]. Kabir AM and colleagues [46] found that *L. salivarius* was against and inhibited colonization of *H. pylori*, when supplemented in eradication therapy may be enhance the eradication of *H. pylori. L. salivarius* inhibited both the attachment and IL-8 release of *H. pylori in vitro*. *H. pylori* could not colonize the stomach of *L. salivarius* infected gnotobiotic BALB/c mice, but colonized in large numbers and subsequently caused active gastritis in germ free mice. In addition, *L. salivarius* given after *H. pylori* implantation could eliminate colonization of *H. pylori* [46]. The conventional meta-analysis by Li S et al. in children demonstrate that probiotics supplementation has efficacy for *H. pylori* eradication therapy [14], which was in line with our conventional meta-analysis; but without more information on specific probiotics. Lately, two network meta-analyses have revealed the comparative effectiveness on probiotics supplementation for *H. pylori* eradication therapy [47, 48]. One network meta-analysis suggested that *L. acidopilus* was a slightly better choice for triple therapy of 7 days and 14 days in adult patients, and *S. boulardii* was more applicable for 10-day triple therapy [48]. Another network meta-analysis of pediatric population suggested that, to supplemented triple therapy, *L. casei* was the best for *H. pylori* eradication rates, and multi-strain probiotics (*L. acidophilus* plus *L. rhamnosus*) for total side effects [47]. These results of above network meta-analyses were different from our study, which might be greatly associated with different population and different area. Additionally, these two network meta-analysis also included some studies from non-Asian counties, which generally used the probiotics of *Lactobacillus* species. However, less studies of *Lactobacillus* species were included in our network meta-analysis, which maybe weaken the strength of evidence on *Lactobacillus* species when ranking the effectiveness among different probiotics.

In 2017, the Joint ESPGHAN/NASPGHAN Guidelines has recommended 14-day triple therapy as first-line therapy for pediatric patients with *H. pylori* infection because of clarithromycin and metronidazole resistances in developing countries [10]. In our study, probiotics, especially *B. infantis+C. butyricum*, as addition could be a new choice for enhancing 14-day triple therapy to eradicate *H. pylori* in children.

Apart from the efficacy of eradication therapy, antibiotic-associated side effects and bacterial resistance cannot be neglected because these factors could lead to the poor tolerability of drugs and hinder patients’ compliance, and consequently cause anti-*H. pylori* treatment failure in clinical practice [49]. In our study, the occurrence of antibiotic-associated total side effects was significantly reduced by the addition of probiotics as well as specific probiotic stains like multi-strain probiotics (such as *B. longum, L. bulgaricus and S. thermophilus*), *S. boulardii* and *L. acidophilus* compared with the placebo (Supplementary Table 2).

This study has several strengths and weaknesses. We concentrated on clinical trials that all investigated14-day triple therapy in Asia where *H. pylori* infection have great impact on children healthy. So, such clinical conclusions for mongoloid race, a main population in Asia, can be firstly obtained. However, the methodologies of all RCTs were not rigorously carried out, which might affect the conclusions. Additionally, strengths of this complex meta-analysis were that the different strains or regimens of probiotics were separated for assessing efficacy and safety. Therefore, probiotics of most effectivity can be guided to add in 14-day triple therapy for the specific purpose, especially to prevent certain side effect. Nevertheless, most trials did not take antibiotic-associated side effects as key outcomes, and not documented more information (such as antibiotic resistant of *H. pylori*, initial treatment or second-line strategy for experiencing a failure of eradicating *H. pylori*, etc.).

All in all, our meta-analyses found that probiotics are beneficial for 14-day triple therapy in Asian pediatric patients with *H. pylori* infection. For obtaining greater benefit of improving eradication rates and reducing antibiotic-associated side effects, the mixture of *B. mesentericus+C. butyricum+S. faecalis* may be recommended to add in 14-day triple therapy. Further direct evidence is needed to warrant it.

## SUPPLEMENTARY MATERIALS FIGURES AND TABLES




