# Supplementary material for: Probiotics in 14-day triple therapy for Asian pediatric patients with Helicobacter pylori infection: a network meta-analysis
Source: Oncotarget. 2017 Oct 7;8(56):96409–18. doi: 10.18632/oncotarget.21633 (PMC5707110; doi:10.18632/oncotarget.21633)
Supplement: Supplementary file 1 [file oncotarget-08-96409-s001.pdf]

Probiotics in 14-day triple therapy for Asian pediatric patients with *Helicobacter pylori* infection: a network meta-analysis

SUPPLEMENTARY MATERIALS

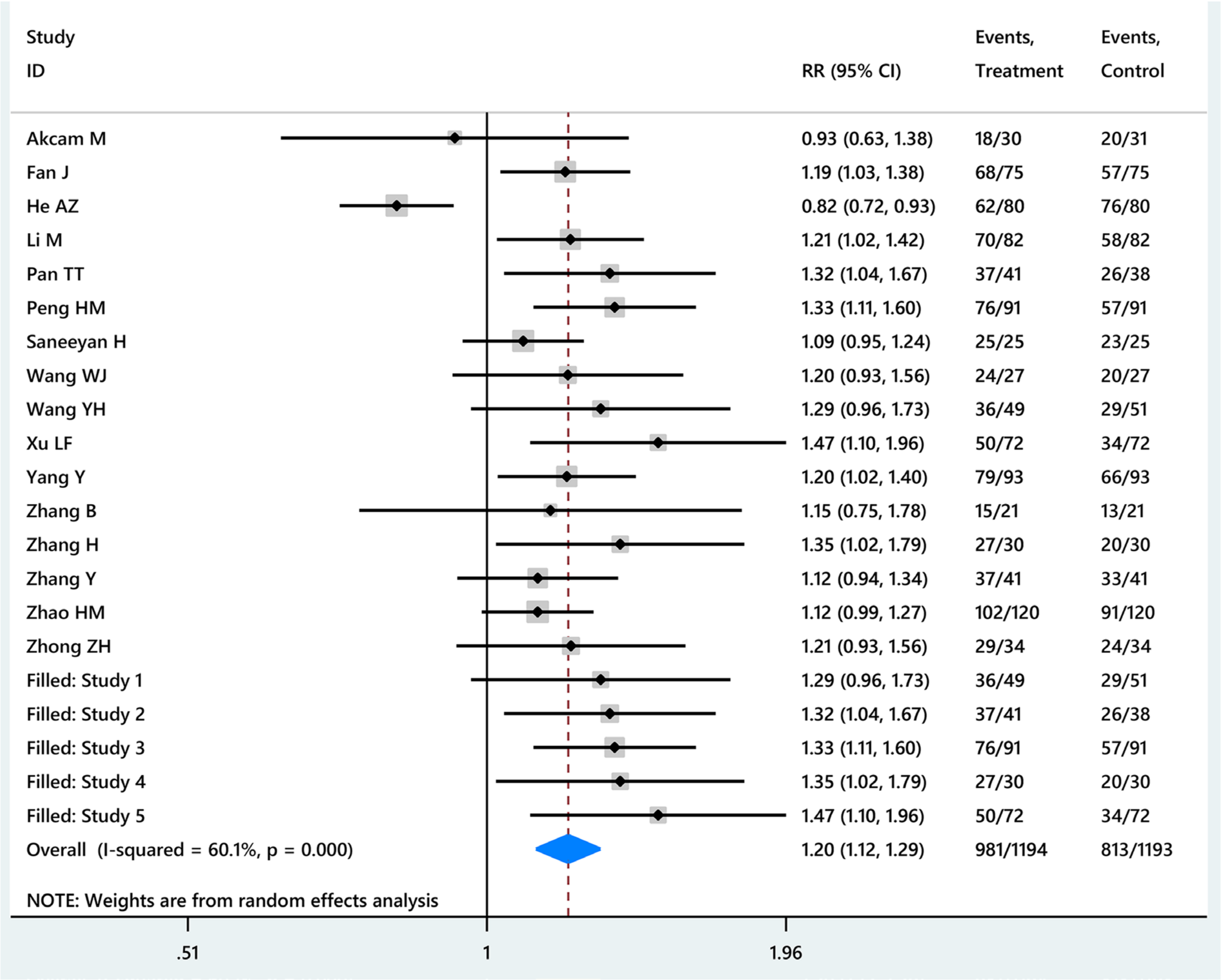

Supplementary Figure 1: Forest plot for the outcome of *Helicobacter pylori* eradication after trimming and inputting 5 studies using the trim and fill method. Multi-strain probiotics 1: *Bifidobacterium animalis+ Lactobacillus acidophilus+Lactobacillus casei;* Multi-strain probiotics 2: *Bacillus mesentericus+Clostridium butyricum+Streptococcus faecalis;* Multi-strain probiotics 3: *Bifidobacterium bifidum+Bifidobacterium infantis+Lactobacillus acidophilus+Lactobasillus bulgaricus+Lactobasillus casei, Lactobasillus reuteri+Streptococcus;* Multi-strain probiotics 4: *Bifidobacterium infantis+Clostridium butyricum;* Multi-strain probiotics 5: *Bifidobacterium longum+Enterococcus faecalis+Lactobacillus acidophilus;* Multi-strain probiotics 6: *Bifidobacterium longum+Lactobacillus bulgaricus+Streptococcus thermophilus;* Multi-strain probiotics 7: *Lactobacillus acidophilus+Bifidobacterium bifidum;* Multi-strain probiotics 8: *Lactobacillus delbrueckii+Lactobacillus acidophilus+Lactococcus lactis*).

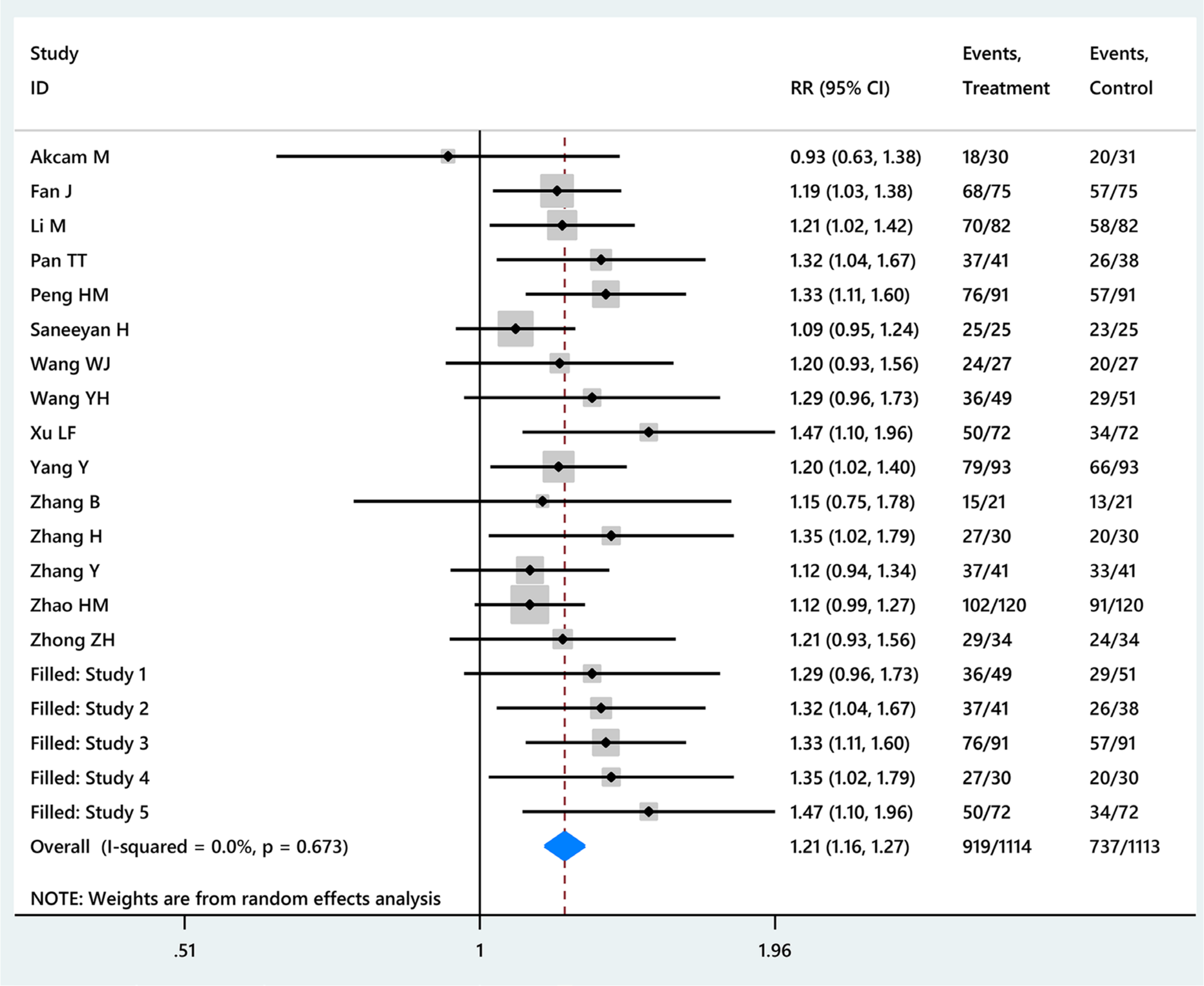

Supplementary Figure 2: Pooled estimate of *Helicobacter pylori* eradication when omitting He AZ et al study on basis of Supplementary Figure 1.

Supplementary Table 1: Conventional meta-analysis based on random effects model for *Helicobacter pylori* eradication of probiotic regimens supplemented 14-day triple therapy compared with placebo

Probiotics VS. Placebo	Number of Studies	Number of Patients	RR (95% CI)	Heterogeneity	
				P Value	I^2(%)
**Total**	**16**	**1822**	**1.16 (1.07–1.26)**	**0.00**	**62.7**
	**15#**	**1662**	**1.19 (1.13–1.25)**	**0.71**	**0**
Bifidobacterium animalis, Lactobacillus acidophilus and Lactobacillus casei	1	61	0.93 (0.63–1.38)	-	-
Bacillus mesentericus, Clostridium butyricum and Streptococcus faecalis	1	182	1.33 (1.11–1.60)	-	-
Bifidobacterium bifidum, Bifidobacterium infantis, Lactobacillus acidophilus, Lactobacillus bulgaricus, Lactobacillus casei, Lactobacillus reuteri and Streptococcus	1	50	1.09 (0.95–1.24)	-	-
Bifidobacterium infantis and Clostridium butyricum	1	144	1.47 (1.10–1.96)	-	-
Bifidobacterium longum, Enterococcus faecalis and Lactobacillus acidophilus	1	186	1.20 (1.02–1.40)	-	-
Bifidobacterium longum, Lactobacillus bulgaricus and Streptococcus thermophilus	4	432	1.08 (0.85–1.36)	0.00	84.6
	3#	272	1.20 (1.07–1.34)	1.00	0
Lactobacillus acidophilus	2	243	1.24 (1.08–1.42)	0.55	0
Lactobacillus acidophilus and Bifidobacterium bifidum	1	100	1.29 (0.96–1.73)	-	-
Saccharomyces boulardii	4	424	1.15 (1.04–1.26)	0.68	0

CI: confidence interval; RR: relative ratio; # indicated that meta-analysis and sensitivity analysis was performed by omitting He AZ (2014).

Supplementary Table 2: Conventional meta-analysis based on random effects model for the safety of probiotic regimens supplemented 14-day triple therapy compared with placebo

Probiotics VS. Placebo	Number of Studies	Number of Patients	RR (95% CI)	Heterogeneity	
				P Value	I^2(%)
**Total side effects**	**12**	**1619**	**0.40 (0.34–0.48)**	**0.87**	**0**
Bacillus mesentericus, Clostridium butyricum and Streptococcus faecalis	1	182	0.31 (0.22–0.45)	-	-
Bifidobacterium infantis and Clostridium butyricum	1	144	0.67 (0.32–1.38)	-	-
Bifidobacterium longum, Enterococcus faecalis and Lactobacillus acidophilus	1	286	0.38 (0.18–0.76)	-	-
Bifidobacterium longum, Lactobacillus bulgaricus and Streptococcus thermophilus	3	278	0.46 (0.35–0.59)	0.93	0
Lactobacillus acidophilus	2	243	0.33 (0.33–0.62)	0.49	0
Lactobacillus acidophilus and Bifidobacterium bifidum	1	100	0.47 (0.18–1.26)	-	-
Lactobacillus delbrueckii, Lactobacillus acidophilus and Lactococcus lactis	1	110	0.28 (0.06–1.32)	-	-
Saccharomyces boulardii	2	276	0.41 (0.24–0.68)	0.89	0

CI: confidence interval; RR: relative ratio.

Supplementary Table 3: League table representing summary estimates on eradication rates of *H. pylori* in network meta-analysis

	**Lactobacillus acidophilus**	**Multi-strain probiotics 2**	**Multi-strain probiotics 4**	**Multi-strain probiotics 5**	**Multi-strain probiotics 6**	**Multi-strain probiotics 7**	**Multi-strain probiotics 8**	**Placebo**	**Saccharomyces boulardii**
Lactobacillus acidophilus		1.06 (0.51–2.21)	0.50 (0.19–1.30)	0.88 (0.34–2.28)	0.73 (0.36–1.45)	0.70 (0.22–2.25)	1.19 (0.22–6.38)	0.33 (0.17–0.62)	0.81 (0.36–1.85)
Multi-strain probiotics 2	0.94(0.45–1.96)		0.47 (0.21–1.06)	0.83 (0.37–1.84)	0.68 (0.43–1.07)	0.66 (0.23–1.87)	1.12 (0.23–5.53)	0.31 (0.22–0.45)	0.76 (0.41–1.44)
Multi-strain probiotics 4	2.02 (0.77–5.32)	2.14 (0.95–4.86)		1.78 (0.64–4.92)	1.47 (0.67–3.19)	1.41 (0.41–4.79)	2.40 (0.43–13.38)	0.67 (0.32–1.38)	1.64 (0.67–4.02)
Multi-strain probiotics 5	1.14 (0.44–2.95)	1.21 (0.54–2.68)	0.56 (0.20–1.56)		0.82 (0.39–1.76)	0.79 (0.24–2.66)	1.35 (0.24–7.46)	0.37 (0.18–0.76)	0.92 (0.38–2.22)
Multi-strain probiotics 6	1.38 (0.69–2.75)	1.46 (0.93–2.30)	0.68 (0.31–1.48)	1.21 (0.57–2.59)		0.96 (0.35–2.66)	1.64 (0.34–7.94)	0.46 (0.35–0.59)	1.12 (0.63–2.00)
Multi-strain probiotics 7	1.43 (0.44–4.62)	1.52 (0.53–4.34)	0.71 (0.21–2.41)	1.26 (0.38–4.24)	1.04 (0.38–2.87)		1.70 (0.27–10.72)	0.47 (0.18–1.26)	1.16 (0.38–3.53)
Multi-strain probiotics 8	0.84 (0.16–4.52)	0.89 (0.18–4.42)	0.42 (0.07–2.32)	0.74 (0.13–4.10)	0.61 (0.13–2.96)	0.59 (0.09–3.70)		0.28 (0.06–1.32)	0.68 (0.13–3.52)
Placebo	3.03 (1.60–5.73)	3.22 (2.23–4.64)	1.50 (0.72–3.11)	2.67 (1.31–5.42)	2.20 (1.69–2.86)	2.11 (0.79–5.64)	3.60 (0.76–17.06)		2.46 (1.47–4.13)
Saccharomyces boulardii	1.23 (0.54–2.80)	1.31 (0.69–2.47)	0.61 (0.25–1.49)	1.08 (0.45–2.61)	0.89 (0.50–1.60)	0.86 (0.28–2.61)	1.46 (0.28–7.54)	0.41 (0.24–0.68)	

Multi-strain probiotics 1: *Bifidobacterium animalis+ Lactobacillus acidophilus+Lactobacillus casei;* Multi-strain probiotics 2: *Bacillus mesentericus+Clostridium butyricum+Streptococcus faecalis*; Multi-strain probiotics 3: *Bifidobacterium bifidum+Bifidobacterium infantis+Lactobacillus acidophilus+Lactobasillus bulgaricus+Lactobasillus casei, Lactobasillus reuteri+Streptococcus;* Multi-strain probiotics 4: *Bifidobacterium infantis+Clostridium butyricum;* Multi-strain probiotics 5: *Bifidobacterium longum+Enterococcus faecalis+Lactobacillus acidophilus;* Multi-strain probiotics 6: *Bifidobacterium longum+Lactobacillus bulgaricus+Streptococcus thermophilus;* Multi-strain probiotics 7: *Lactobacillus acidophilus+Bifidobacterium bifidum;* Multi-strain probiotics 8: *Lactobacillus delbrueckii+Lactobacillus acidophilus+Lactococcus lactis*).

Supplementary Table 4: League table representing summary estimates on total side effects in network meta–analysis

	Lactobacillus acidophilus	Multi-strain probiotics 1	Multi-strain probiotics 2	Multi-strain probiotics 3	Multi-strain probiotics 4	Multi-strain probiotics 5	Multi-strain probiotics 6	Multi-strain probiotics 7	Placebo	Saccharomyces boulardii
Lactobacillus acidophilus		1.35 (0.79–2.31)	0.94 (0.62–1.42)	1.16 (0.79–1.70)	0.85 (0.53–1.36)	1.05 (0.70–1.56)	1.18 (0.88–1.58)	0.97 (0.61–1.56)	1.25 (0.98–1.60)	1.07 (0.79–1.45)
Multi-strain probiotics 1	0.74 (0.43–1.27)		0.70 (0.39–1.25)	0.86 (0.49–1.51)	0.63 (0.34–1.18)	0.78 (0.44–1.38)	0.87 (0.52–1.45)	0.72 (0.38–1.35)	0.93 (0.58–1.50)	0.79 (0.48–1.33)
Multi-strain probiotics 2	1.06 (0.70–1.60)	1.43 (0.80–2.57)		1.23 (0.78–1.92)	0.91 (0.54–1.53)	1.11 (0.70–1.77)	1.25 (0.86–1.82)	1.03 (0.61–1.74)	1.33 (0.96–1.86)	1.14 (0.78–1.66)
Multi-strain probiotics 3	0.86 (0.59–1.27)	1.17 (0.66–2.06)	0.81 (0.52–1.27)		0.74 (0.45–1.22)	0.91 (0.59–1.40)	1.02 (0.72–1.44)	0.84 (0.51–1.39)	1.09 (0.8–1.47)	0.93 (0.65–1.32)
Multi-strain probiotics 4	1.17 (0.73–1.87)	1.58 (0.85–2.96)	1.10 (0.66–1.86)	1.36 (0.82–2.24)		1.23 (0.74–2.05)	1.38 (0.89–2.13)	1.14 (0.64–2.01)	1.47 (0.99–2.2)	1.26 (0.81–1.95)
Multi-strain probiotics 5	0.95 (0.64–1.42)	1.29 (0.72–2.29)	0.90 (0.57–1.42)	1.10 (0.71–1.71)	0.81 (0.49–1.36)		1.12 (0.78–1.61)	0.93 (0.55–1.55)	1.20 (0.87–1.65)	1.02 (0.71–1.48)
Multi-strain probiotics 6	0.85 (0.63–1.14)	1.15 (0.69–1.91)	0.80 (0.55–1.16)	0.98 (0.70–1.39)	0.72 (0.47–1.12)	0.89 (0.62–1.28)		0.82 (0.53–1.28)	1.07 (0.90–1.26)	0.91 (0.71–1.17)
Multi-strain probiotics 7	1.03 (0.64–1.65)	1.39 (0.74–2.60)	0.97 (0.57–1.64)	1.19 (0.72–1.97)	0.88 (0.5–1.55)	1.08 (0.65–1.81)	1.21 (0.78–1.88)		1.29 (0.86–1.93)	1.10 (0.71–1.72)
Placebo	0.80 (0.63–1.02)	1.08 (0.67–1.74)	0.75 (0.54–1.05)	0.92 (0.68–1.24)	0.68 (0.46–1.02)	0.84 (0.61–1.15)	0.94 (0.79–1.11)	0.77 (0.52–1.16)		0.85 (0.71–1.03)
Saccharomyces boulardii	0.93 (0.69–1.26)	1.26 (0.75–2.10)	0.88 (0.60–1.28)	1.08 (0.76–1.53)	0.80 (0.51–1.24)	0.98 (0.68–1.41)	1.10 (0.86–1.41)	0.91 (0.58–1.41)	1.17 (0.98–1.41)	

Multi-strain probiotics 1: *Bifidobacterium animalis+ Lactobacillus acidophilus+Lactobacillus casei;* Multi-strain probiotics 2: *Bacillus mesentericus+Clostridium butyricum+Streptococcus faecalis*; Multi-strain probiotics 3: *Bifidobacterium bifidum+Bifidobacterium infantis+Lactobacillus acidophilus+Lactobasillus bulgaricus+Lactobasillus casei, Lactobasillus reuteri+Streptococcus;* Multi-strain probiotics 4: B*ifidobacterium infantis+Clostridium butyricum;* Multi-strain probiotics 5: *Bifidobacterium longum+Enterococcus faecalis+Lactobacillus acidophilus;* Multi-strain probiotics 6: B*ifidobacterium longum+Lactobacillus bulgaricus+Streptococcus thermophilus;* Multi-strain probiotics 7: *Lactobacillus acidophilus+Bifidobacterium bifidum;* Multi-strain probiotics 8: *Lactobacillus delbrueckii+Lactobacillus acidophilus+Lactococcus lactis.*

Supplementary Table 5: Characteristics of included studies in the meta-analysis. See Supplementary_Table_5
