# Supplementary material for: Probiotics in 14-day triple therapy for Asian pediatric patients with Helicobacter pylori infection: a network meta-analysis
Source: Oncotarget. 2017 Oct 7;8(56):96409–18. doi: 10.18632/oncotarget.21633 (PMC5707110; doi:10.18632/oncotarget.21633)
Supplement: Supplementary file 2 [file oncotarget-08-96409-s002.docx]

| **Supplementary Table 5: Characteristics of included studies in the meta-analysis** | | | | | | | | | | | | |
| --- | --- | --- | --- | --- | --- | --- | --- | --- | --- | --- | --- | --- |
| Study | Year | Region | Study design | Complains or diagnosis | Patients' characteristics | | | | Treatment arms | | | Jadad |
|  |  |  |  |  |  |  |  |  | Eradication regimens | Probiotics regimens | |  |
|  |  |  |  |  | No. of patients | Diagnosis of H. pylori infection | Confirmation of eradication | Follow-up | Antibiotics | Probiotics | Doses  (CFU/per day) |  |
| Akcam M | 2015 | Turkey | Single center, RCT, open study | Uninvestigated dyspepsia, with predominantly chronic or recurrent upper abdominal pain | 61 | RUT + histology | UBT | 6 weeks | lansoprazole 30 mg, qd, amoxicillin 50 mg/kg/day, clarithromycin 15 mg/kg/day, bid | *Bifidobacterium animalis, Lactobacillus acidophilus and Lactobacillus casei* | 1.4*10^10 | 2 |
| Fan J | 2016 | China | Single center, RCT, open study | Abdominal pain, nausea and vomiting, bloating or anorexia | 150 | UBT | UBT | 4 weeks | omeprazole 0.7-0.8 mg/kg, bid, amoxicillin 50 mg/kg, tid, clarithromycin, 15 mg/kg, bid | *Bifidobacterium longum, Lactobacillus bulgaricus and Streptococcus thermophilus* | 4.5 g | 3 |
| He AZ | 2014 | China | Single center, RCT, open study | Peptic ulcers or chronic gastritis | 160 | UBT + histology | UBT | 4 weeks | omeprazole 0.8 mg/kg/day, 2 of (amoxicillin 30-50 mg/kg/day, or clarithromycin 15 mg/kg/day, or metronidazole 7.5-10 mg, tid) | *Bifidobacterium longum, Lactobacillus bulgaricus and Streptococcus thermophilus* | 4.5 g | 2 |
| Li M | 2014 | China | Single center, RCT, open study | Peptic ulcers or chronic gastritis | 164 | UBT + histology | na | 2 weeks | esomeprazole, 20mg, bid, amoxicillin 1g,qd, clarithromycin 500 mg, bid | *Lactobacillus acidophilus* | 3 g | 3 |
| Pan TT | 2010 | China | Single center, RCT, open study | Peptic ulcers or chronic gastritis | 79 | histology + (UBT/RUT) | UBT | 4 weeks | omeprazole 0.8 mg/kg, amoxicillin 50 mg/kg, clarithromycin 15 mg/kg, qd | *Lactobacillus acidophilus* | 10^10 | 2 |
| Pan XL | 2015 | China | Single center, RCT, open study | Chronic gastritis | 110 | UBT | UBT | 4 weeks | rabeprazole, 10 mg, bid, amoxicillin 500 mg, bid, furazolidone 100 mg, bid | *Lactobacillus delbrueckii, Lactobacillus acidophilus and Lactococcus lactis* | 1.98 g | 2 |
| Peng HM | 2015 | China | Single center, RCT, open study | Chronic gastritis | 182 | RUT + UBT | UBT | 4 weeks | omeprazole 0.6-0.8 mg/kg, qd, amoxicillin 50 mg/kg, bid, clarithromycin 15-30 mg/kg, bid | *Bacillus mesentericus, Clostridium butyricum and Streptococcus faecalis* | 600 mg | 2 |
| Saneeyan H | 2011 | Iran | Single center, RCT, double-blind, placebo-controlled | Symptoms (such as abdominal pain, bloating, vomitting, anaemia, etc.) | 50 | histology +UBT | UBT | 4 weeks | omeprazole 0.5 mg/kg, amoxicillin 25 mg/kg, clarithromycin 10 mg/kg, bid | *Bifidobacterium bifidum, Bifidobacterium infantis, Lactobacillus acidophilus, Lactobacillus bulgaricus, Lactobacillus casei, Lactobacillus reuteri and Streptococcus* | 10^9 | 3 |
| Wang WJ | 2011 | China | Single center, RCT, open study | Chronic abdominal pain | 54 | UBT | UBT | 4 weeks | omeprazole 0.6 mg/kg, amoxicillin 25 mg/kg, clarithromycin 10 mg/kg, bid | *Bifidobacterium longum, Lactobacillus bulgaricus and Streptococcus thermophilus* | 3*10^8 | 3 |
| Wang YH | 2014 | China | Single center, RCT, open study | Peptic ulcers | 100 | UBT | UBT | 6 weeks | PPI 0.6-0.8 mg/kg, clarithromycin 10-15 mg/kg, amoxicillin 30–50 mg/kg, and metronidazole (15–20 mg/kg for penicillin allergic children), bid | *Lactobacillus acidophilus and Bifidobacterium bifidum* | 4 g | 3 |
| Xu LF | 2016 | China | Single center, RCT, open study | Abdominal pain, belching, bloating or acid reflux | 144 | UBT | UBT | 4 weeks | omeprazole 0.8-1.0 mg/kg, bid, amoxicillin 50 mg/kg, bid, clarithromycin 15 mg/kg, bid | *Bifidobacterium infantis and Clostridium butyricum* | 1680 mg | 3 |
| Yang Y | 2013 | China | Single center, RCT, open study | Peptic ulcers or chronic gastritis | 186 | UBT + histology | na | 2 weeks | esomeprazole 20mg, bid,amoxicillin 1g, qd, clarithromycin 500 mg, bid | *Bifidobacterium longum, Enterococcus faecalis and Lactobacillus acidophilus* | 6 g | 3 |
| Zhang B | 2015 | China | Single center, RCT, open study | Chronic abdominal pain or chronic vomiting | 194* | ELISA(IgG)/histology | UBT | 4 weeks | omeprazole, clarithromycin, amoxicillin or omeprazole, clarithromycin, metronidazole (na dose) | *Saccharomyces boulardii* | 500 mg | 3 |
| Zhang H | 2013 | China | Single center, RCT, open study | Chronic gastritis | 60 | RUT + UBT | UBT | 4 weeks | omeprazole 0.6 mg/kg, clarithromycin 15 mg/kg, amoxicillin 50 mg/kg, qd | *Saccharomyces boulardii* | 500 mg | 2 |
| Zhang Y | 2012 | China | Single center, RCT, open study | Peptic ulcers or chronic gastritis | 82 | 2 of (UBT, histology and RUT) | UBT | 4 weeks | omeprazole 0.8 mg/kg, amoxicillin 50 mg/kg, clarithromycin 15 mg/kg, qd | *Saccharomyces boulardii* | 250 mg | 2 |
| Zhao HM | 2014 | China | Single center, RCT, open study | Children with esophagitis, gastric or duodenal tumor and corrosive gastritis or duodenitis, were excluded. | 240 | UBT + histology | UBT | 4 weeks | omeprazole 0.7-0.8 mg/kg/day, qd, amoxicillin 40 mg/kg/day, tid, clarithromycin 15 mg/kg/day, bid | *Saccharomyces boulardii* | 500 mg | 3 |
| Zhong HZ | 2015 | China | Single center, RCT, open study | Peptic ulcers or chronic gastritis | 68 | UBT | UBT | 1 month | omeprazole 20 mg, qd/bid, amoxicillin 1000 mg, bid, furazolidone 100 mg, bid | *Bifidobacterium longum, Lactobacillus bulgaricus and Streptococcus thermophilus* | 1260 mg | 2 |
| * Indicated that 42 of 194 patients reported eradication data. Abbreviations: UBT: urea breath test, RUT: rapid urease test, HpSA: *H. pylori* stool antigen, ELISA: enzyme-linked immunosorbent assay, CFU: colony-forming units. | | | | | | | | | | | | |
